# A novel *PHEX* mutation associated with vitamin D-resistant rickets

**DOI:** 10.1038/s41439-019-0040-3

**Published:** 2019-02-14

**Authors:** Saori Sako, Yo Niida, Kosuke Robert Shima, Yumie Takeshita, Kiyo-aki Ishii, Toshinari Takamura

**Affiliations:** 1 Department of Endocrinology and Metabolism, Kanazawa University Graduate School of Medical Sciences, 13-1 Takara-machi, Kanazawa, Ishikawa 920-8640 Japan; 2 Division of Genomic Medicine, Department of Advanced Medicine, Medical Research Institute, Kanazawa Medical University, Ishikawa, 920-0293 Japan

**Keywords:** Osteogenesis imperfecta, Osteogenesis imperfecta

## Abstract

X-linked hypophosphatemic rickets (XLH) is the most common form of hereditary rickets. Here, we present a case of XLH associated with a novel mutation in a phosphate-regulating gene with homologies to endopeptidases on the X chromosome (*PHEX*). PCR-direct sequencing revealed a novel *PHEX* mutation in exon 22, NM_000444.6(*PHEX*):c.2202del [p.Asn736Ilefs*4], near the 3′-UTR region encoding the COOH-terminal extracellular domain. In silico analysis indicated that a single mutation in N736 may have caused a significant change in higher-order protein structure and function.

X-linked hypophosphatemic rickets (XLH), the most common form of hereditary rickets, is associated with impaired renal tubular resorption that causes chronic hypophosphatemic rickets. A 2010 national epidemiological survey in Japan reported an estimated incidence rate of XLH of approximately 1 in 20,000^1^. Hypophosphatemic rickets includes various diseases, such as vitamin D metabolite dysfunction, renal tubular abnormality, fibroblast growth factor 23 (FGF23)-related hypophosphatemia, and phosphorus deficiency. FGF23, which comprises 251 amino acids, decreases the expression of type 2a and type 2c sodium-phosphorus cotransporters, suppresses proximal renal tubular phosphorus reabsorption, and concurrently alters the expression of vitamin D metabolizing enzyme, resulting in a decrease in the concentration of 25-(OH)_2_^2^. FGF23-related hypophosphatemia is caused by genetic mutations in phosphate-regulating gene with homologies to endopeptidases on the X chromosome (*PHEX*)^3^, *FGF23*, dentin matrix protein 1 (*DMP1*), ectonucleotide pyrophosphatase/phosphodiesterase (*ENPP1*), and family with sequence similarity 20, member C (*FAM20C*). *PHEX* comprises 22 exons, encodes a type 2 transmembrane protein with a single-pass structure and is primarily expressed in bones but not the kidneys^4^. Since a *PHEX* gene mutation was first identified in 1995, at least 364 mutations, including nonsense mutations, have been registered in the Human Gene Mutation Database (http://www.hgmd.cf.ac.uk/ac/index.php). However, although *PHEX* mutations are thought to increase the production of FGF23, which promotes the phosphorus diuretic effect, resulting in hypophosphatemia, how *PHEX* gene mutations increase FGF23 and which domain is responsible for the function of *PHEX* remain unknown^5^. Therefore, understanding the relationship between mutation loci in *PHEX* and heterogeneous clinical features of XLH is necessary to better understand the function of PHEX in regulating FGF23 and phosphorus metabolism.

A 40-year-old Japanese woman was referred to our hospital for a detailed examination of her rickets. She had a short stature and bow legs but lacked a family history of congenital diseases and short stature. She had been born at full term via vaginal delivery. At 1 year of age, she experienced gait disturbance and was diagnosed with vitamin D-resistant rickets. Despite treatment with 1,25-(OH)_2_D_3_, her bone lesions worsened. At 25 years of age, her height was 131 cm. X-ray examinations revealed genu varum and Looser’s zones in the tibias. She underwent surgeries for bone correction and hip adductor muscle dissection. When the patient was 40 years of age, we performed laboratory tests, X-ray examinations of the lower limbs, computed tomography (CT) of the cervical spine, and measurements of bone mineral density (BMD).

Table 1 presents laboratory test data for the patient on admission. She had a phosphate (Pi) level of 1.8 (normal range, 2.5–4.5) mg/dL, a calcium (Ca) level of 9.0 (normal range, 8.0–10.5) mg/dL, an alkaline phosphatase (ALP) level of 255 (normal range, 115–359) IU/L, a bone-type ALP level of 82.4%, an intact parathyroid hormone (PTH) level of 62.4 (normal range, 10.3–65.9) pg/mL, a 25-hydroxyvitamin D3 [25-(OH)D_3_] level of 12.0 (normal range, 7–41) ng/mL, a 1,25-(OH)_2_D_3_ level of 47.2 (normal range, 20–60) pg/mL, an FGF23 level of 42 (normal range, <30) pg/mL, a tubular maximum phosphate reabsorption per glomerular filtration rate of 1.88 (normal range, 2.3–4.3) mg/dL, and an urine Ca level of 0.116 (normal range, 0.1–0.3) g/day.

**Table 1:** Laboratory data of the patient on admission

	Patient	Reference range
P (mg/dL)	1.8	2.5–4.5
Ca (mg/dL)	9	8.0–10.5
ALP (IU/L)	255	115–359
25-(OH)D_3_ (ng/mL)	12	7–41
1.25-(OH)_2_D_3_ (pg/mL)	47.2	20–60
Intact PTH (pg/mL)	62.4	10–65
BAP (g/L)	23.6	2.9–22.6
FGF23 (pg/mL)	42	<30
TmP/GFR (mg/mL)	1.88	2.3–4.3
TRP (%)	92	81–90
Estimated GFR (ml/min per 1.73 m^2^)	161.89	≧90
Urine Ca (g/day)	0.116	0.1–0.3

X-ray examinations revealed genu varum and Looser’s zones in the tibias, and cervical spine CT revealed calcification of the posterior longitudinal ligament of the spine with no evidence of tumors. Her BMD was 1.694 g/cm^2^ at the lumbar spine (L2–L4).

Based on the findings of bone changes, hypophosphatemia, and elevated FGF23 levels, FGF23-related hypophosphatemia rickets was suspected; consequently, a genetic analysis was performed. Genomic DNA was extracted from whole blood samples using a genomic DNA extraction kit (Qiagen, Hilden, Germany). PCR-direct sequencing revealed a *PHEX* mutation, NM_000444.6(*PHEX*):c.2202del [p.Asn736Ilefs*4], in exon 22 (Fig. 1). This mutation was not found in various databases, including the Genome Aggregation Database (http://gnomad.broadinstitute.org), the Exome Aggregation Consortium database (http://exac.broadinstitute.org), and the Human Gene Mutation Database, suggesting that this mutation is novel rather than an SNP. Therefore, the patient’s definitive diagnosis was XLH caused by a novel *PHEX* mutation. The mutation is believed to be “de novo”, as assessed from a clinical perspective. She was treated with activated vitamin D metabolites and phosphate salts.

**Fig. 1:**
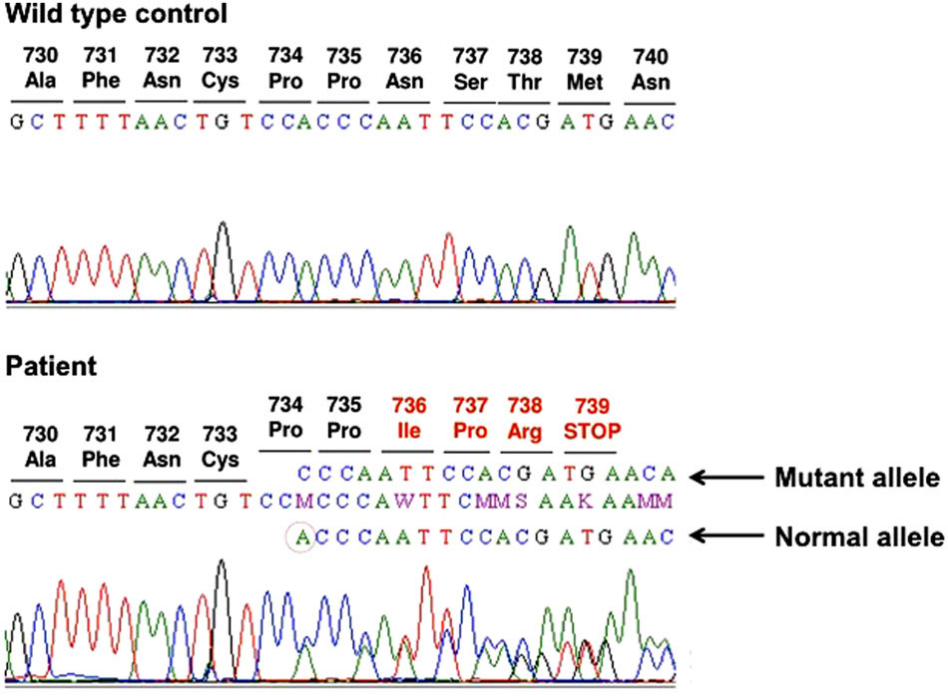
Genomic DNA sequence of the patient’s *PHEX* gene, indicating a novel mutation, NM_000444.6(*PHEX*):c.2202del [p.Asn736Ilefs*4], in exon 22

To our knowledge, we have described the first reported case of XLH caused by NM_000444.6(*PHEX*):c.2202del [p.Asn736Ilefs*4] in exon 22. This single base deletion induces a premature stop codon at position 739. Although this *PHEX* gene mutation is located near the 3′-UTR region encoding the COOH-terminal extracellular domain, which contains no putative zinc binding sites or active sites, we believe that this mutation causes XLH based on the following reasons. First, the deleted region of the *PHEX* gene is highly conserved among wide ranges of mammalian species, with homology of >88% for the 17 C-terminal amino acids in humans, mice, rats, rabbits, cats, dogs, horses, pigs, and bats. Furthermore, p.C733, p.C746, p.R747^6^, and p.N736 (our case), which are mutated in patients with XLH, are completely conserved across species. These results suggest that this region is critical for the function of the protein. Second, we predicted the impact of this novel mutation on protein structure by conducting in silico analyses using software tools, including PROVEAN (http://provean.jcvi.org/index.php) and PANTHER (http://pantherdb.org/about.jsp); these in silico prediction engines produced scores of −4.43 and −3.05, respectively, both of which suggested deleteriously damaged PHEX function. Third, in two other cases, XLH was reported to be associated with missense mutations located in the COOH-terminal region of the *PHEX* gene (C746W and R747X)^6^. Fourth, it is conceivable that even a single mutation causing a premature stop codon eliminates mRNA transcripts via the mechanism known as nonsense-mediated mRNA decay. Finally, our patient did not have any mutations in the genes encoding DMP1 and ENPP1, which are also involved in FGF23-related hypophosphatemia. In any event, because the protein encoded by the mutated *PHEX* is unknown, the causal phenotypic role of this mutation in rickets requires future study.

A limitation of the present report is lack of investigation of the mutant PHEX protein observed in the patient. As has been previously reported, it is necessary to investigate the trafficking, endopeptidase activity, and conformation of this protein^7^.

In summary, a single N736 mutation in the COOH-terminal extracellular domain of PHEX may result in a significant change in higher-order protein structure and function and may cause XLH by increasing the production of FGF23. We believe that the present report contributes to understanding the relationship between mutation loci in the *PHEX* gene and heterogeneous clinical features of XLH.

## Data Availability

The relevant data from this Data Report are hosted at the Human Genome Variation Database at 10.6084/m9.figshare.hgv.2522
